# Organ damage evaluation in a temperature-controlled circulatory arrest rat model

**DOI:** 10.1186/s12872-022-02955-5

**Published:** 2022-12-06

**Authors:** Yaoyao Xiong, Quan Zheng, Chunle Wang, Wei Yan, Wei He

**Affiliations:** 1 Extracorporeal Life Support Center of Cardiovascular Surgery, The Second Xiangya Hospital, Central South University, Changsha, 410011 China; 2 Department of Urology, Xiangya Hospital, Central South University, Changsha, 410008 China

**Keywords:** Cardiopulmonary bypass, Deep hypothermic circulatory arrest (DHCA), Animal model, Temperatures

## Abstract

**Background:**

Deep hypothermic circulatory arrest (DHCA) is commonly used in adult aortic surgery and pediatric complex congenital heart disease, and is associated with pathophysiological changes and postoperative complications. Here, a temperature-controlled circulatory arrest model in rats was established to study the suitable temperature of circulatory arrest by investigating the damage to body organs under different temperatures.

**Methods:**

Thirty Sprague‒Dawley rats were randomly divided into 5 equal groups for DHCA experiments: I (15–20 °C), II (20–25 °C), III (25–30 °C), IV (normothermic cardiopulmonary bypass), and V (sham operation group). Blood gas analysis, homodynamic parameters, and intervals of cardiac recovery were measured at different time points in all groups. Morphological changes in intestinal tissue were observed under light and electron microscopes. Oxidative stress was measured by MPO activity, MDA, and SOD content. Tissue damage was confirmed by serum detection of ALT, AST, BUN, Cr, and LDH. To examine the inflammatory response, cytokines, including IL-1, IL-4, IL-10, IFN-γ, and TNF-α, were detected.

**Results:**

The extracorporeal circulation technique caused damage to the body; the degree of the damage caused by the circulatory arrest technique may be related to circulating temperature, with the least amount of damage occurring at 20–25 °C compared to 15–20 °C and 25–30 °C. Ischemia and hypoxia can cause intestinal tissue damage, which manifests primarily as a loss of the intestinal mucosal barrier. Ischemic intestinal damage caused by DHCA was not associated with inflammation.

**Conclusion:**

The study provides new insights into the pathophysiologic mechanisms of DHCA.

## Background

Cardiopulmonary bypass (CPB) replaces the function of the heart and lungs while the heart is arrested for providing a bloodless, stable surgical field [1]. Cardiopulmonary bypass technology has been routinely applied to many heart surgeries [2]. Deep hypothermic circulatory arrest (DHCA) is a kind of CPB widely used in clinical surgeries, such as acute aortic dissection or aortic surgery for infants and young children with complex congenital heart disease [3, 4]. However, CPB provides a clear surgical field for heart surgeries, and it also causes some damage to patients with cardiac surgery simultaneously [5]. In-depth studies of the process of cardiopulmonary bypass, especially the pathophysiological changes during the deep hypothermic circulatory process in the study, are imminent. This requires establishing simple animal models for in-depth study of the deep hypothermic circulatory process. Extracorporeal circulation is a nonphysiological process but not a simple disease process. During cardiopulmonary bypass, the body undergoes blood dilution, hypothermia, nonpulsatile perfusion, ischemia–reperfusion injury, the release of inflammatory cytokines due to foreign body contact, and an abnormal state of the coagulation system, most of which could simply be explained by pathophysiology [6, 7]. Even the pharmacokinetics of the drug in the cardiopulmonary bypass process also need to be re-evaluated and considered due to the alternation of physical conditions.

Cardiopulmonary bypass is used not only in the field of cardiovascular surgery but also in other fields, such as acute respiratory distress syndrome (ARDS) treatment [8], liver transplantation, brain surgery, urology, general surgery, and even in vitro hyperthermia treatment of hepatitis C [9], cancer, and AIDS [10]. However, cardiopulmonary bypass also has drawbacks and limitations, the main drawbacks being foreign body contact, heparin and blood damage [11]. Postoperative complications caused by cardiopulmonary bypass include neurological complications [12, 13], such as cerebral ischemia and hypoxia injury, cerebral infarction and intracranial hemorrhage; circulatory complications [13], such as postoperative low cardiac output and refractory arrhythmia; complications such as atelectasis and acute respiratory distress syndrome [14]; blood system complications such as heparin-induced thrombocytopenia syndrome and hemorrhage [15, 16]; urinary complications such as acute renal insufficiency [17]; digestive system complications [18, 19]; and gastrointestinal bleeding [20–22].

With the development of cardiovascular surgery, the cardiopulmonary bypass has also ushered in a new era. Cardiac surgery methods continue to be updated and upgraded, and the development of cardiopulmonary bypass technology has brought new challenges. High-quality cardiopulmonary bypass escort makes complex cardiac surgery possible. DHCA has been increasingly applied in the clinic, such as in adult aortic dissection for large vascular surgery and infants and young children with complex congenital correction of heart disease surgery [3, 23]. There is currently a "rewarming" trend in cardiopulmonary bypass. That is, the temperature of cardiopulmonary bypass surgery is constantly improving, and many heart centers are exploring the use of room temperature cardiopulmonary bypass technology and have achieved great success [24]. Therefore, it is of great clinical significance to explore circulatory temperature.

In the past, people were concerned about damage to the nervous system, especially the brain, in the DHCA process, so the deep hypothermia low flow anterograde/retrograde cerebral perfusion came into being. Experimental studies are often concentrated on the nervous system, while few studies and research have investigated the damage to the extracorporeal circulation of other organs, especially the organ damage in cardiopulmonary bypass surgery. In this study, we established a rat model of circulatory arrest at different temperatures and performed a detailed investigation of organ damage under different temperatures during DHCA. We believe our findings will shed new light on the pathophysiological mechanisms underlying DHCA.

## Materials and methods

### Equipment

Microsurgical, surgical microscopy and conventional surgical instruments were purchased from the Carl Zeiss Company (Germany). A respiratory anesthesia machine and a small animal ventilator were purchased from Surgivet and Harvard Apparatus (USA). The Powerlab multichannel physiological monitor was a product of AD Instruments Corporation (USA). The blood gas analyzer was a product of the NOVA Biomedical company (USA). A stocker double head roller pump was purchased from Stockert company (Germany). The variable temperature blanket was from ATC1000 (Sarasota, USA). The Jostra HCU20-600 variable temperature water tank was from the Maquet Critical Care AB, Solna, Sweden. Special small animal lung membranes were produced by Dongguan Kewei Medical Supplies Ltd. (China). The thermostat was purchased from Xijing Medical Supplies Co., Ltd. (China). The anesthesia trocar (16G, 20G, 22G) was from United States Becton Dickinson company. Venous drainage tube syringes (1 ml, 2 ml, 5 ml, 20 ml) were obtained from Beijing Freseniuskabi Pharmaceutical Co., Ltd. (China). The surgical suture was from Johnson & Johnson (USA). The glass thermometer and the Flow cytometry Model LSR II were from BD (Biosciences; USA). Desktop Centrifuge was purchased from Beckman Coulter (USA). The electronic balance was purchased from the Beijing Sartorius company. The − 80 °C and − 20 °C refrigerants were obtained from the Germany SIEMENS company. The thermostatic temperature water bath was from Shanghai Yuejin Medical Instrument Factory (China). The biological tissue paraffin embedding machine and Stapler were purchased from the Leica company (Germany). The single-channel micropipettes were from Gilson (Germany). The spectrophotometer was from Shanghai Tianpu Analytical Instrument Co., Ltd. (China). The microplate reader and SoftMax Pro microplate reading software SpectraMaxPlus384 were from BD, USA. The swirling Mixer was from Lin Bell Instrument Manufacturing Co., Ltd. (China). The BX51 optical microscope and DP71 camera system were from Olympus Corporation (Japan). The JSM-5500 scanning electron microscopy was from JEOL Co., Ltd. (Japan). The Hitachi Model H-7650 transmission electron microscope was from Hitachi (Japan). The TEM imaging system was from AMT imaging (MA, USA). The glass slide for fixed tissues was from Wuhan Boster Biological Technology Co. (China). The EP tube, tissue homogenizer, frozen tube, biochemical blood tubes, and test tube liquid nitrogen biological containers were from Wuhan Boster Biological Technology Co. (China).

### Materials

6% HES130/0.4 was purchased from Beijing Fresenius Kabi Pharmaceutical Co., Ltd. (China). Heparin was obtained from Changzhou Qianhong Biochemical Pharmaceutical Co., Ltd. (China). The myeloperoxidase (MPO), superoxide dismutase (SOD), and malondialdehyde (MDA) assay kits were from the Nanjing Jiancheng Research Institute of Bioengineering (China). The BD CBA Mouse/Rat Soluble Protein Master Buffer Kit, Rat IL-10 Flex Set (A8), IL-1a Flex Set (A4), IL-4 Flex Set (B9), IFN-y Flex Set (A6), TNF Flex Set (C8) and the BD CBA Mouse/Rat Soluble Protein Master Buffer Kit were purchased from BD (USA).

### Animals

Thirty healthy adult Sprague‒Dawley rats weighing approximately 350–450 g were provided by SLAC Laboratory Animal Company (Changsha, China) and kept with clean grade and feeding conditions according to the SPF standard. Before the experiment, rats received 12 h of fluorescence/12 h of dark room temperature in the animal feeding room in the Central South University Animal Center. The experimental study was approved by the Institutional Animal Care and Use Committee (IACUC) of The Second Xiangya Hospital, Central South University. All rats were sacrificed by cervical dislocation to collect intestinal tissue for follow-up experiments.

### Surgical procedures

The rats were fasted for 12 h with free access to water before surgery. The rats were randomly selected and weighed. Before the operation, the time point T1 was recorded. For surgical procedures, the rats were first inducted for inhalation anesthesia with sevoflurane and then restrained. To monitor the rectal temperature, the thermometer lubricated by paraffin oil was inserted into the anal cavity. The rats were anesthetized with 100% pure oxygen mixed with 3% sevoflurane using a 16 G anesthesia cannula. The initial respiratory frequency was set at 60 beats/min, and the tidal volume was 8–10 ml/kg, which can be adjusted according to the blood gas value. After the operation, the rats were anesthetized by mechanical ventilation. Three vascular puncture tubes were punctured into the tail artery, left femoral artery, and right jugular vein under the surgical microscope operation. The left femoral artery was used for continuous hemodynamic monitoring during the bypass procedure; the caudal artery was used to connect the arterial end of the extracorporeal circuit, and the artery was perfused; the right jugular vein was connected to the venous drainage tube to the venous end of the extracorporeal circuit. The venous blood of the rats was drained. The left femoral artery was punctured with a 22 G trocar and 400 IU/kg heparin to heparinize the rat; the caudal artery was punctured with a 20 G cannula and connected to the perfusion end of the extracorporeal circulation; intravenous catheterization of the right jugular vein was performed using a modified 22 G IV silicone tube. Fine surgical scissors were used to cut the jugular vein. Under the guidance of the arterial sheath guidewire, the right jugular vein was drained into the right jugular vein. The distance was determined and labeled in the experiment), and the gravity height of venous drainage was 30–35 cm. All the distal vessels of the vessels were ligated with silk thread, and the proximal cardiac thread was fixed to prevent slipping. After the extracorporeal circulation pipeline connection, the crystal liquid was precharged with exhaust gas, and colloidal liquid (6% HES130/0.4) was used to replace the crystal liquid, keeping the liquid level at 1–2 ml. This model is a bloodless prefilled model. After catheterization, the venous drainage tube was placed in the reflux chamber, and the pliers were opened to start the cardiopulmonary bypass. To maintain the backroom fluid surface balance, the main pump diversion speed was adjusted. A membrane lung oxygen-air mixture of oxygen was used to maintain the flow of gas during the bypass of PaCO_2_ and PaO_2_ in the normal range. When the blood pressure dropped during the bypass, the liquid level could not be maintained, so more liquid was added to keep the blood pressure and fluid colloid level stable. There were no vasoactive drugs used during the entire experimental process. The temperature of the rats was lowered to the target temperature by using a variable temperature water tank, and the expected cooling time was 30 min.

Stopping cycle: cooling to the target temperature before the preparation of the prestop recording time T2; after stopping the venous end to continue drainage, take 20 ml syringe from the reflux chamber out of rat blood storage until the blood pressure reaches a straight line and the rat heart stops beating only. The cycle time was maintained for 30 min, and the cycle was stopped if the body temperature increased. The surface of the body evenly crushed ice to maintain the temperature.

Rewarming stage: at the beginning of cardiopulmonary bypass, the syringe was slowly reinjected into the blood chamber. Then, the temperature to rectal temperature was increased to 35 degrees Celsius, the rewarming time was expected to be 30 min, the temperature to the rectal temperature was approximately 35 degrees Celsius, and the rewarming time was 30 min. However, the rewarming time was kept as long as possible within one hour according to the actual situation. When the heart resumes, the time point T3 is recorded.

Stopping stage: After the temperature reached the target temperature, the machine was stopped, and the time point T4 was recorded before stopping. 5. After the operation, the ventilator was maintained for 60 min after cardiopulmonary bypass, and the time point T5 was recorded.

### Serum specimen

A total of 3 ml of whole blood from rats was extracted at the T1 time point, and 5 ml of whole blood from rats were extracted at the T5 time point. Whole blood was collected, placed in a 5 ml centrifuge tube, and placed on ice. After centrifuging at 3000 rpm for 15 min, the upper serum was kept at − 80 °C until use.

### Tissue preparation

The intestine samples were cut to a 5 mm × 5 mm × 5 mm size and fixed into 10% formalin fixative (phosphate buffer pH 7.0) for hematoxylin–eosin (HE) staining. The intestine samples were quickly put into the precooled balanced salt solution; a 2 mm × 2 mm × 2 mm tissue sample was placed in 4% glutaraldehyde fixed for electron microscopy scanning or fixed with 1% osmium tetroxide for scanning electron microscopy scanning. The remaining parts were packed in cryopreserved tubes, frozen in liquid nitrogen, and stored at − 80 °C for further experiments.

### Serum biochemical marker analysis

The serum levels of alanine aminotransferase (ALT), aspartate aminotransferase (AST), creatinine (Cr), blood urea nitrogen (BUN), and lactate dehydrogenase (LDH) were measured by an automatic biochemical analyzer.

### Transmission electron microscope and scanning electron microscope analysis

For transmission electron microscope analysis, the fixed tissue samples were embedded in Spurr resin and sliced using an ultramicrotome (LEICA EM UC7). The samples were then stained with uranyl acetate and alkaline lead citrate (each for 5 to 10 min) and observed in a Hitachi Model H-7650 TEM with AMT TEM imaging system. For scanning electron microscope analysis, the fixed tissue samples were dehydrated and dried, covered with gold and examined under JSM-5500 scanning electron microscope.

### HE staining

The fixed specimens were paraffin-embedded and sliced to a thickness of 5 μm. Then, the slices were stained with hematoxylin and eosin, dehydrated with ethanol, cleared with xylene, sealed with neutral gum, and covered with a coverslip for the optical microscope observation.

### Tissue SOD, MPO, and MDA activity detection

The tissue homogenate diluted with saline buffer was centrifuged at 2500 r/min for 10 min, and then the subsequent activity detection assay was carried out. The enzyme activity was determined according to the ELISA kit. The SOD activity unit is regarded as 50% inhibition of SOD per mg of tissue protein in a 1 ml reaction. For MPO, the enzyme activity unit was calculated as the decomposition of 1 mmol of H_2_O_2_ per gram of wet tissue at 37 °C in the reaction system.

### Cytokine detection

The cytokines from each sample were detected using a Cytometric Bead Array (BD) and analyzed using a BD LSR II flow cytometer. The standard operation was carried out according to the instructions.

### Experimental data acquisition

The blood pressure and heart rate of the rats were monitored continuously by LabChart7.0 software (AD Instruments Co., Ltd.); the rats were infused with 1 ml of heparin sodium salt water at the five time points T1, T2, T3, T4 and T5. Arterial blood samples were taken from the rats for blood gas analysis.

### Statistical analysis

Statistical analysis was performed using GraphPad Prism 5.0 software. All data were analyzed by one-way ANOVA/two-way ANOVA followed by post hoc Tukey's test. *P* < 0.05 was considered to be statistically significant.

## Results

### Establishment of a temperature-controlled circulatory arrest model

**Fig. 1:**
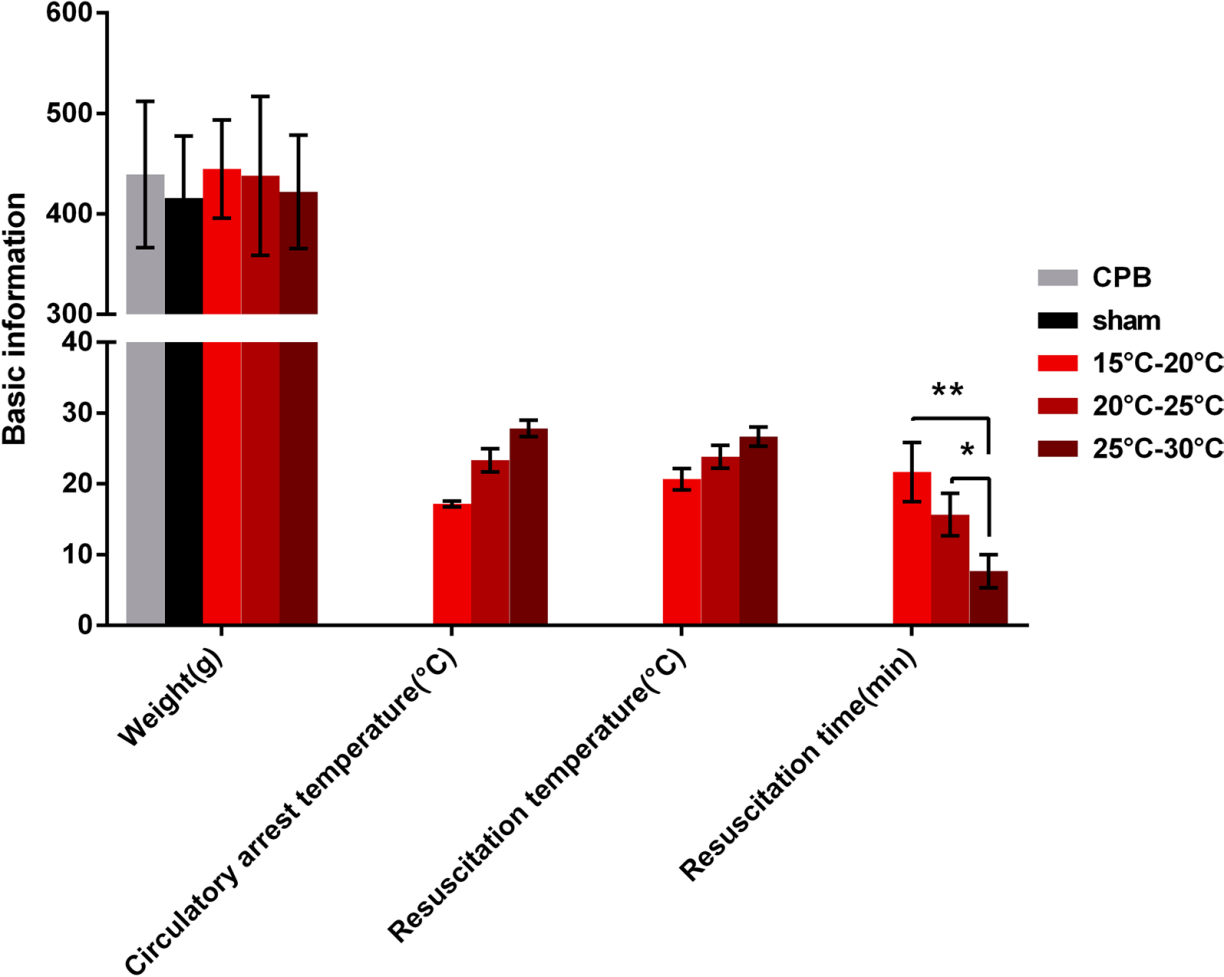
Basic information about the animals used in the temperature-controlled circulatory arrest experiments, including the average animal weight (**A**), circulatory arrest temperature (**B**), resuscitation temperature (**C**), and resuscitation time (**D**). Group I (15–20 °C), group II (20–25 °C), group III (25–30 °C), group IV (normal CPB group), and group V (sham operation group). The data are expressed as the mean ± SD from three independent experiments. The differences between groups were evaluated using one-way ANOVA. n = 6, *P < 0.05, **P < 0.01 compared to the CPB group

The basic information of rats showed that the weight of rats from different groups were similar. There are no significant difference of circulatory arrest temperature and resuscitation temperature among group I, II and III. While, the resuscitation time was increased in group I (I> II> III, *P* < 0.05) (Fig. 1). During the whole experiment, the pH value, PaO_2_ and PaCO_2_ of the rats in each group were maintained within the normal range (Tables 1 and 2). There was no significant difference in mean arterial pressure (MAP) between the experimental groups (Table 3). During the process of temperature rewarming, the temperature had a significant impact on the blood pressure. The MAP of groups I, II and III was decreased at T2 and T3, and the trend was statistically significant (*P* < 0.05). The MAP in group V was significantly higher than that in groups I, II, III and IV (*P* < 0.05). Additionally, there was no significant difference in heart rate (HR) between the experimental groups (Table 4). During the experiment, the HR of the rats decreased; the HR of groups I, II and III also decreased, but the differences were not statistically significant (*P* > 0.05). At T5, the IV and V groups had significantly higher HR than the I, II, and III groups. During the whole experiment, the lactic acid value of group V remained virtually unchanged. There was no significant difference in the lactic acid values among the I, II, III and IV groups at the T2 time point (*P* > 0.05) (Table 5). At T3 and T4, the lactic acid values of groups I, II, III and IV were significantly higher than those of group V (III  > I >  II  > IV, *P* < 0.05). After 60 min (T5), the lactic acid values of the IV group remained higher than those of the V group, despite a significant decrease. The lactic acid value of the II group decreased most significantly, and there was no significant difference among groups I, II, III and IV (*P* > 0.05) (Table 5). The hematocrit (HCT) was maintained above 20%, and the Hb was maintained above 6 g/dL (Tables 6 and 7).

**Table 1:** The Mean pH values of the blood in the experimental groups

Parameters	T1	T2	T3	T4	T5
PH					
I (15 °C–20 °C)	7.33 ± 0.14	7.42 ± 0.20	7.44 ± 0.25	7.45 ± 0.20	7.37 ± 0.09
II (20 °C–25 °C)	7.43 ± 0.08	7.55 ± 0.04	7.40 ± 0.23	7.43 ± 0.14	7.36 ± 0.07
III (25 °C–30 °C)	7.42 ± 0.11	7.62 ± 0.05	7.43 ± 0.11	7.45 ± 0.14	7.33 ± 0.19
IV (normal CPB)	7.42 ± 0.04	7.59 ± 0.05	7.55 ± 0.06	7.58 ± 0.11	7.46 ± 0.08
V (sham)	7.42 ± 0.02	7.41 ± 0.06	7.41 ± 0.06	7.44 ± 0.05	7.48 ± 0.06

The differences between groups were evaluated using two-way ANOVA. There was no significant difference between the different groups and the IV group. There was no significant difference between the different time points and the T1 time points

**Table 2:** The mean partial pressure of oxygen (PaO_2_) in the experimental groups

Parameters	T1	T2	T3	T4	T5
PaO_2_ (mmHg)					
I (15 °C–20 °C)	426.13 ± 58.32	490.53 ± 80.92	403.6 ± 50.70	3455.8 ± 46.20	414.03 ± 45.33
II (20 °C–25 °C)	450.78 ± 57.00	398.9 ± 64.86	380.4 ± 45.65	396.16 ± 23.81	421.12 ± 40.61
III (25 °C–30 °C)	457.04 ± 58.36	40.101 ± 66.21	407.04 ± 52.13	407.11 ± 81.47	389.9 ± 61.48
IV (normal CPB)	429.6 ± 39.60	419.2 ± 53.10	384.65 ± 43.98	396.5 ± 38.51	458.3 ± 93.40
V (sham)	413.85 ± 77.68	434.25 ± 27.52	504.73 ± 87.29	480.73 ± 22.95	516.45 ± 98.11

The differences between groups were evaluated using two-way ANOVA. There was no significant difference between the different groups and the IV group. There was no significant difference between the different time points and the T1 time points

**Table 3:** The mean arterial pressure (MAP) between the experimental groups

Parameters	T1	T2	T3	T4	T5
MAP (mm Hg)					
I (15 °C–20 °C)	114.86 ± 15.77	90.26 ± 10.74*	94.78 ± 14.94*	110.36 ± 13.12	113.85 ± 12.43
II (20 °C–25 °C)	114.46 ± 20.09	88.64 ± 14.74*	89.99 ± 13.8**	95.57 ± 16.4	97.99 ± 14.89
III (25 °C–30 °C)	119.26 ± 19.87	93.81 ± 14.20*	94.31 ± 14.65*	112.66 ± 10.74	120.28 ± 11.28
IV (normal CPB)	110.89 ± 17.69	120.07 ± 10.88	123.30 ± 17.03	104.82 ± 16.62	110.24 ± 8.40
V (sham)	140.39 ± 25.74	149.96 ± 21.80	146.64 ± 26.82	148.17 ± 24.20	149.00 ± 26.82

The differences between groups were evaluated using two-way ANOVA. *P < 0.05, **P < 0.01, T2, T3 time points between the different groups with the IV group were different; no significant difference between different time points in each group and T1 time points

**Table 4:** The mean heart rate (HR) between the experimental groups

Parameters	T1	T2	T3	T4	T5
HR (time/min)					
I (15 °C–20 °C)	338.37 ± 35.34	307.97 ± 26.72	307.94 ± 12.77	310.4 ± 21.98	304.09 ± 14.70
II (20 °C–25 °C)	345.18 ± 46.88	310.58 ± 25.70	304.88 ± 57.90	309.93 ± 54.14	313.06 ± 39.60
III (25 °C–30 °C)	325.82 ± 22.51	284.53 ± 51.63	294.31 ± 42.44	276.81 ± 41.89	276.51 ± 41.92
IV (normal CPB)	338.48 ± 34.76	332.28 ± 45.52	329.53 ± 41.24	329.04 ± 43.72	332.42 ± 52.89
V (sham)	359.29 ± 39.38	352.48 ± 37.94	349.84 ± 45.16	342.66 ± 19.91	345.23 ± 53.29

The differences between groups were evaluated using two-way ANOVA. There was no significant difference between the different groups and the IV group. There was no significant difference between different time points and the T1 time points

**Table 5:** The mean lactic acid (Lac) levels in the experimental groups

Parameters	T1	T2	T3	T4	T5
Lac (mmol/L)					
I (15 °C–20 °C)	0.76 ± 0.42	1.89 ± 0.9^#^	8.01 ± 0.44^**##^	7.53 ± 0.62^**##^	3.98 ± 0.35^##^
II (20 °C–25 °C)	0.83 ± 0.33	1.94 ± 0.97^#^	10.56 ± 0.90^**##^	10.2 ± 0.37^**##^	2.92 ± 0.72^##^
III (25 °C–30 °C)	1.06 ± 0.65	2.90 ± 1.07^##^	13.20 ± 0.39^**##^	10.87 ± 0.91^**##^	3.63 ± 0.84^##^
IV (normal CPB)	0.92 ± 0.31	2.83 ± 0.53^##^	5.3 ± 0.46^##^	5.22 ± 0.596^##^	3.05 ± 0.62^##^
V (sham)	0.95 ± 0.41	0.80 ± 0.24	0.83 ± 0.12	0.78 ± 0.10	0.93 ± 0.05

The differences between groups were evaluated using two-way ANOVA. * For different groups compared with the IV group, *P < 0.05, **P < 0.01; # indicates each group at different time points compared with the T1 time points, #*P* < 0.05, ##*P* < 0.01

**Table 6:** The mean hematocrit (HCT) in different experimental groups

Parameters	T1	T2	T3	T4	T5
HCT(%)					
I (15 °C–20 °C)	38.63 ± 2.88	23.14 ± 4.14	20.57 ± 3.91	21.71 ± 4.03	26.50 ± 4.76
II (20 °C–25 °C)	37.50 ± 2.56	22.43 ± 2.64	22.00 ± 2.62	21.00 ± 1.83	25.8 ± 3.27
III (25 °C–30 °C)	37.00 ± 2.08	20.86 ± 1.86	20.71 ± 2.36	17.86 ± 3.29	22.17 ± 3.37
IV (normal CPB)	35.33 ± 2.42	22.00 ± 2.45	20.60 ± 2.70	21.67 ± 3.27	24.00 ± 2.45
V (sham)	38.50 ± 2.38	35.50 ± 2.38	37.00 ± 3.61	33.75 ± 2.87	34.75 ± 3.59

The differences between groups were evaluated using two-way ANOVA. There was no significant difference between the different groups and the IV group. There was no significant difference between the different time points and the T1 time points

**Table 7:** The mean hemoglobin (Hb) concentrations in experimental groups

Parameters	T1	T2	T3	T4	T5
Hb (g/dl)					
I (15 °C–20 °C)	12.85 ± 0.95	7.73 ± 1.41	6.90 ± 1.35	7.26 ± 1.3	8.85 ± 1.30
II (20 °C–25 °C)	12.48 ± 0.91	7.49 ± 0.89	7.29 ± 0.86	6.98 ± 0.67	8.58 ± 1.05
III (25 °C–30 °C)	12.34 ± 0.66	6.97 ± 0.60	6.84 ± 0.80	6.01 ± 1.02	7.35 ± 1.13
IV (normal CPB)	11.78 ± 0.77	7.33 ± 0.85	7.18 ± 1.11	7.17 ± 1.03	8.05 ± 1.16
V (sham)	12.75 ± 0.83	11.85 ± 0.60	12.3 ± 1.18	11.2 ± 0.94	11.63 ± 1.18

The differences between groups were evaluated using two-way ANOVA. There was no significant difference between the different groups and the IV group. There was no significant difference between the different time points and the T1 time points

### Morphological changes in intestinal tissue after DHCA

**Fig. 2:**
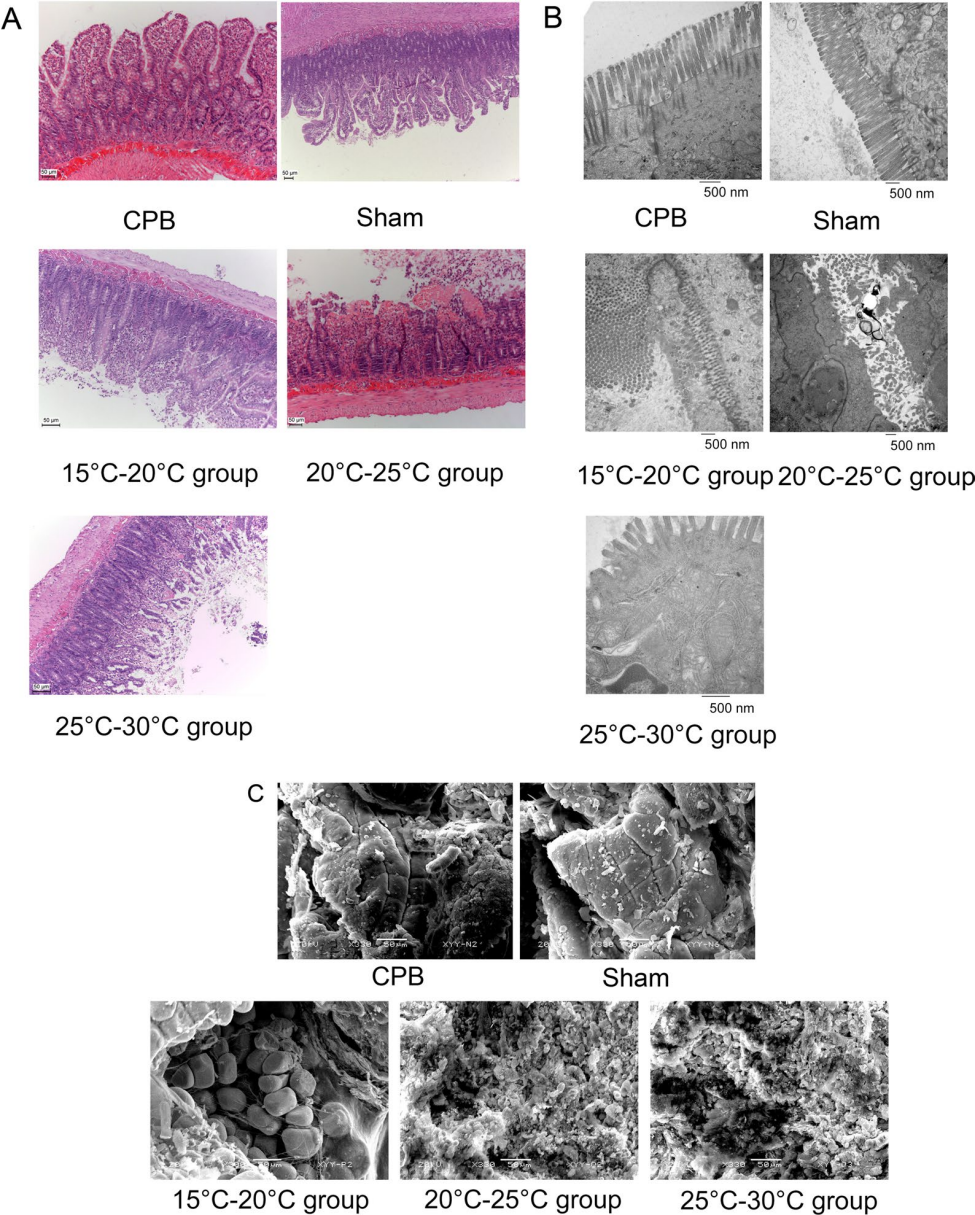
The microstructure of the intestine specimens from each experimental group was observed by HE staining (**A**), transmission electron microscopy (**B**), and electron microscopy (**C**). The integrity of the intestinal mucosal epithelial cell structure, swelling of the mitochondria, mitochondrial crest, and cytoplasm within the rough endoplasmic reticulum structure were analyzed. Group I (15–20 °C), group II (20–25 °C), group III (25–30 °C), group IV (normal CPB group), and group V (sham operation group). The data are expressed as the mean ± SD from three independent experiments. **A**, **C** scale bar is 50 μm, resolution is 300 dpi; B scale bar is 500 nm, resolution is 1307 dpi. No downstream processing was performed in the images

In the sham group (group V), the intestinal mucosa was intact, the intestinal villi were neat, there was no top epithelial exfoliation, and no subepithelial space was widened. Layer integrity was intact, there was no bleeding or congestion, and the glands were nearly normal. In the CPB group (room temperature), the intestine was intact, intestinal villi were arranged neatly, intestinal villi topped the subepithelial space, and occasionally the gap was expanded. Layer integrity was intact, there was no bleeding or congestion, and the glands were nearly normal. In Group I (15–20 °C), the intestinal mucosa was intact, intestinal villi were arranged neatly, intestinal villi exhibited epithelial degeneration and necrosis, and cortical epithelial separation was observed with missing villus tops. Layer integrity was intact, with minimal bleeding or congestion, and the glands were nearly normal. However, in group II (20–25 °C), the intestinal integrity was intact, intestinal villi disappeared, there was lamina propria dilatation with vascular bare, there was visible bleeding and congestion, and the glands were nearly normal. In group III (25–30 °C), intestinal integrity was intact, intestinal villi disappeared, and the inherent layer of ulcers disintegrated. There was considerable bleeding and congestion, and the glands were nearly normal (Fig. 2A). Similar results were observed under the electron microscope (Fig. 2B, C). In the CPB group (IV), microvilli were neatly aligned and slightly loose. The columnar epithelium was intact, there was no mitochondrial swelling, and the mitochondrial ridge arrangement was normal. Glands with normal lamina propria. Group I (15–20 °C) exhibited microvilli loss and fracture. The columnar epithelium was intact, the mitochondria were enlarged and the mitochondria ridge arrangement was normal. Glands with normal lamina propria. In group II (20–25 °C), microvilli fractures and deletions were observed. The columnar epithelium was intact, the mitochondria were swollen, and the mitochondrial ridges were absent. Glands with normal lamina propria. In group III (25–30 °C), there were microvilli fractures and deletions. The columnar epithelium was intact, the mitochondria were swollen, and the mitochondrial ridges disappeared. Glands with normal lamina propria. These results indicate that the degree of intestinal tissue damage varies with temperature.

### Blood biochemical results

**Fig. 3:**
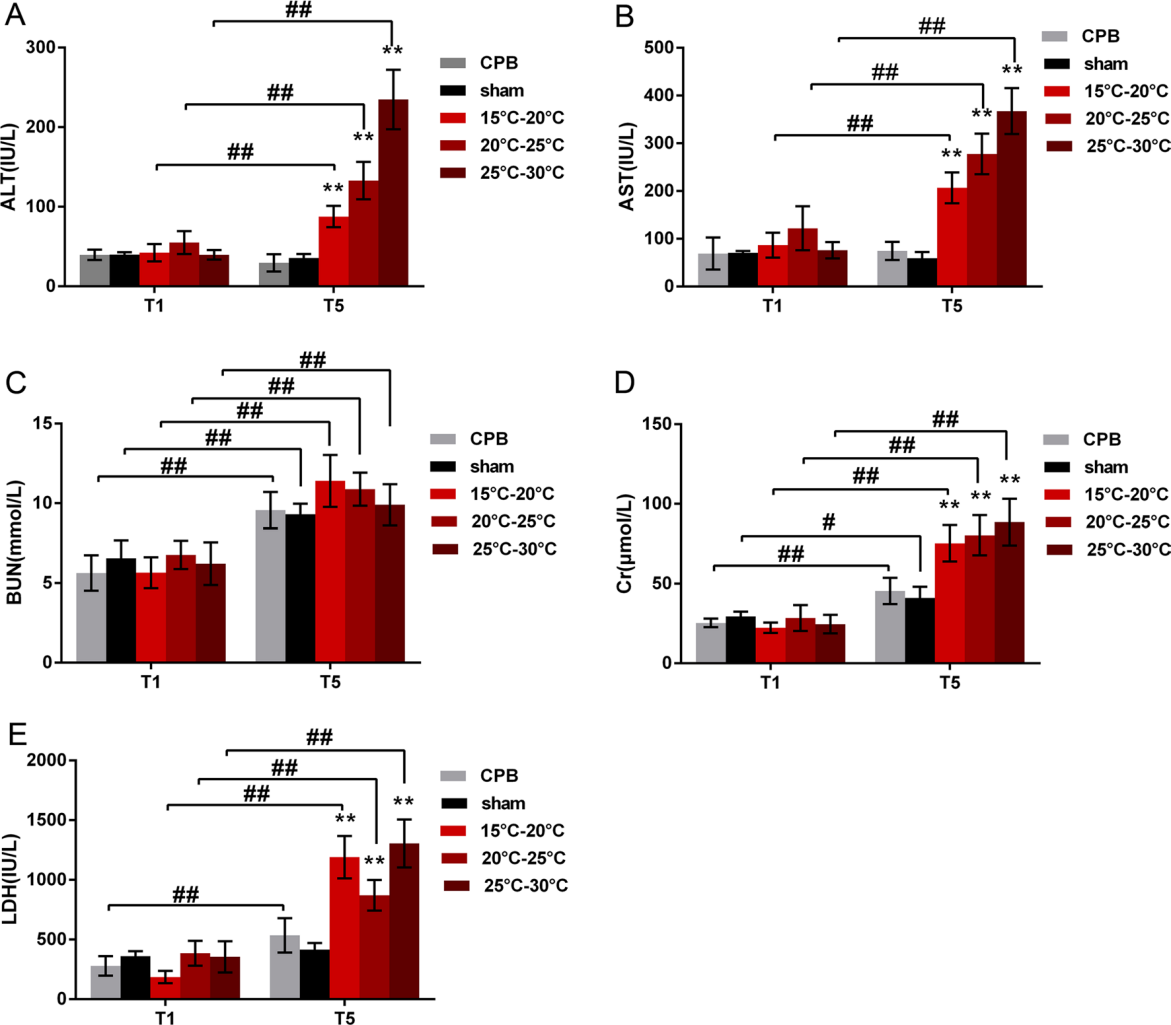
The levels of the biochemical blood markers; alanine aminotransferase (ALT) (**A**), aspartate transaminase (AST) (**B**), blood urea nitrogen (BUN) (**C**), creatinine (Cr) (**D**), and lactate dehydrogenase (LDH) (**E**) were measured in each experimental group. The data are expressed as the mean ± SD from three independent experiments. The differences between groups were evaluated using two-way ANOVA. n = 6, **P < 0.01 compared to the CPB group, ##*P* < 0.05, ##*P* < 0.01 compared to T1

We measured the ALT and AST levels in serum to examine liver function. As a result, we found no significant difference in ALT and AST between the five experimental groups at the T1 time point (*P* = 0.96 and 0.52). At the T5 time point, the levels of ALT and AST in all rats in the three circulatory arrest groups increased compared to those in the CPB and sham operation groups (*P* < 0.01). In groups I, II and III, the ALT and AST values were significantly increased after the operation (T5) compared with before the operation (T1) (*P* < 0.01) (Fig. 3A, B). To test kidney function, we examined BUN and serum Cr. Before bypass, the BUN levels in the five experimental groups were not significantly different at the T1 time point (*P* = 0.47). At the T5 time point, the BUN levels of the five experimental groups were increased, but there was no significant difference among the five groups (*P* = 0.5) (Fig. 3C). In the serum Cr levels, there was no significant difference between the five experimental groups before bypassing at T1 (*P* = 0.78). Meanwhile, at the T5 time point, the serum Cr levels of the five experimental groups were increased (*P* < 0.05 or 0.01). Moreover, the serum Cr levels increased significantly in groups I, II, and III compared to the CPB (IV) and sham operation (V) groups (*P* < 0.01) (Fig. 3D). To examine heart function, we evaluated LDH activity and found no difference at the T1 time point (*P* = 0.78), but the LDH indexes of groups I, II, III and IV were increased at the T5 time point (*P* < 0.01) (Fig. 3E).

### Expression of inflammatory cytokines

**Fig. 4:**
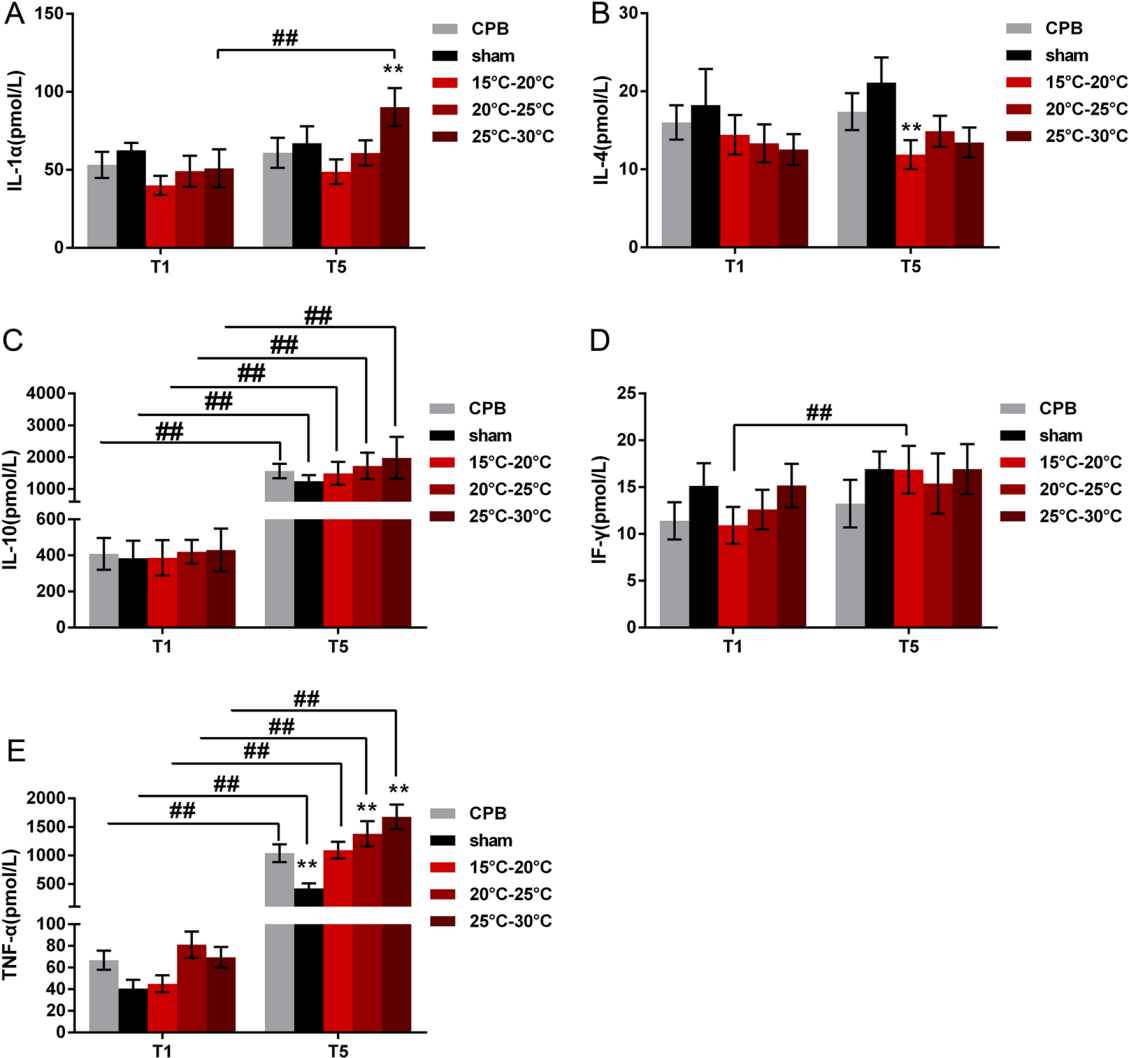
Inflammatory cytokine release in each experimental group. The concentrations of IL-1α (**A**), IL-4 (**B**), IL-10 (**C**), IFN-γ (**D**), and TNF-α (**E**) were examined. Group I (15–20 °C), group II (20–25 °C), group III (25–30 °C), group IV (normal CPB group), and group V (sham operation group). The data are expressed as the mean ± SD from three independent experiments. The differences between groups were evaluated using two-way ANOVA. n = 6, **P < 0.01 compared to the CPB group, ##*P* < 0.01 compared to T1

There was no significant difference in the levels of IL-1α, IL4, IL10, INF-γ or TNF-α among the five experimental groups before bypass (*P* > 0.05). At the T5 time point, the value of IL-1α in group III increased (*P* < 0.01) (Fig. 4A). The IL-4 values of all groups did not change significantly at T5 compared to T1. At the T5 time point, the IL-4 levels were significantly decreased in group I compared to the CPB (IV) group (Fig. 4B). The IL-10 levels were significantly increased in all the groups at T5 compared to T1. However, there was no significant difference among the five groups (*P* > 0.05) (Fig. 4C). Compared with T1, the IFN-γ value in group I (15–20 °C) increased at T5 (*P* < 0.01). The other four groups were not significantly different between the T1 and T5 time points (*P* > 0.05) (Fig. 4D). The TNF-α levels in groups II, III and IV were significantly higher than those before the operation (T1) (*P* < 0.05) (Fig. 4E).

### Oxidative stress examination

**Fig. 5:**
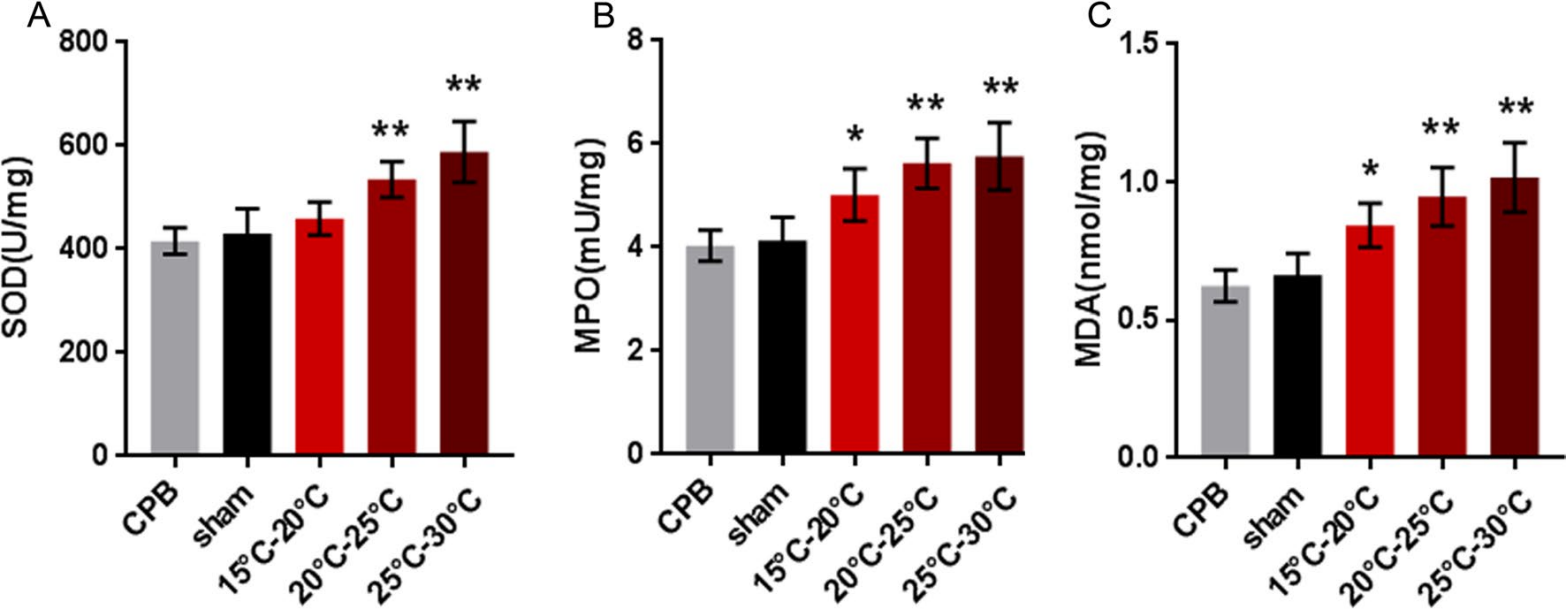
Evaluation of oxidative stress markers in the small intestine. The superoxide dismutase (SOD) (**A**), myeloperoxidase (MPO) (**B**), and malondialdehyde (MDA) (**C**) activities in each organ were examined. Group I (15–20 °C), group II (20–25 °C), group III (25–30 °C): group IV (normal CPB group), group V (sham operation group). The data are expressed as the mean ± SD from three independent experiments. The differences between groups were evaluated using one-way ANOVA. n = 6, *P < 0.05, **P < 0.01, compared to the CPB group

First, we analyzed SOD and MPO activity in the small intestine. We found that SOD activity in group II (20–25 °C) and group III (25–30 °C) was higher than that in the other three groups (*P* < 0.05) (Fig. 5A). In addition, we also found that MPO activity in the intestine of the rats in group I (15–20 °C), group II (20–25 °C) and group III (25–30 °C) was higher than that in the control (*P* < 0.05) (Fig. 5B). Subsequently, we also examined the MDA content in tissues. Similarly, we found that the intestinal MDA of rats in groups I, II and III was higher than that in the CPB and sham operation groups (*P* < 0.05 or 0.01) (Fig. 5C). These results indicate that the intestine is very sensitive to oxidative stress during circulatory arrest.

## Discussion

The use of DHCA in clinical practice has continued to increase in recent years. Therefore, the establishment of a simple and easy-to-use model of DHCA in vitro has been a hot topic of interest for researchers, and most of the previous animal models of in vitro circulation are large and medium-sized animals, such as rabbits, sheep, dogs, and pigs [25–27]. These animals have the advantage of being more anatomically similar to humans, which facilitates. the simulation of clinical procedures. However, these animals are expensive, and molecular biology is scarce, making the investigation of cellular and molecular mechanisms difficult. The advantages of using rats to establish animal models are as follows: 1) the anatomy of the cardiovascular system in rats is similar to that of humans; 2) the rat model is an in vivo model, and its results are more convincing when compared to the ex vivo model; 3) the molecular biology assays in rats are abundant and diverse and have a higher homology with humans; and 4) the cost of animals is low and economical. In this experiment, we successfully established an ex vivo rat circulatory model for stopping circulation at different temperatures without blood precharging. In comparison to previously reported rat models [28–31], this model employs the method of draining blood from the rat to halt circulation by rendering the heart ischemic. Temperature regulation is fast and precise, and the overall experimental procedure is safe and feasible, which has a positive significance for studying the effects of stopping circulation on different organs of the body under different temperatures.

There is a low incidence of digestive complications following extracorporeal circulation, but the mortality rate is high [32–35]. Our preliminary experimental results show that intestinal injury could occur after deep hypothermia stopped circulating, indicating that DHCA, a special mode of extracorporeal circulation, can cause severe damage to the gastrointestinal tract. In the present study, several biochemical indicators were examined in DHCA experiments using SD rats; the results showed that cardiopulmonary bypass could cause small intestinal injury in rats, and temperature-dependent stop-circulation could exacerbate the injury. The 25–30 °C stop-circulating group had the most severe organ damage, followed by the 15-20ºC stop-circulating group, and the visceral damage was the least severe in the 20–25 °C stop-circulating group. Although there is no comparable literature reporting temperature-related injury after DHCA, previous authors reported that rapid rewarming after DHCA in rats under strict avoidance of hyperthermia was accompanied by significant histological damage and exacerbation of NF-κB expression in the brain [36]. Therefore, the temperature and speed of rewarming after DHCA may be factors influencing the injury.

Disruption of the intestinal mucosal barrier, ischemia–reperfusion injury, inflammatory response, and ischemia-hypoxia due to hypoperfusion may be the mechanisms of injury to the gastrointestinal system triggered by deep hypothermic cessation of blood flow. The traditional mechanism of intestinal injury suggests that the permeability of the intestine is greatly increased after extracorporeal circulation, allowing for bacterial colonization of the intestine, which leads to the release of inflammatory factors into the bloodstream, resulting in sepsis, among other complications, in patients [37, 38]. However, in the present study, there were no significant differences in inflammatory factors in the five experimental groups. During cardiopulmonary bypass, many factors, including foreign body contact, ischemia‒reperfusion injury [39, 40], and endotoxin effects [41], can contribute to the development of inflammatory responses. The lack of a statistically significant difference in the current results may be due to the following factors: (1) incorrect measurement time and extracorporeal circulation led to an increased release of inflammatory factors, but the release did not reach the peak, therefore the results were not statistically significant. (2) Extracorporeal circulation can cause the release of inflammatory factors, which may be related to stopping circulation; the temperature of stopping circulation had little effect on the release of inflammatory factors. (3) Current research indicates that extracorporeal circulation following intestinal complications and the release of inflammatory factors and the development of an inflammatory response are inconsistent [42, 43], suggesting that the mechanism of cardiopulmonary bypass after gastrointestinal injury and inflammation may not be related.

Although the techniques and methods used in the present study are all indicative of a sophisticated investigation to answer the challenging question of the optimal temperature of DHCA, there are limitations to our research. Even though the rat model of CPB and DHCA was established to closely resemble clinical standards, median sternotomy, direct cardiac cannulation, and surgical intervention were not performed. These factors may have a remarkable effect on the degree of inflammation and tissue marker serum levels. These issues should be addressed in our future research.

## Conclusion

The study provides new insights into the pathophysiologic mechanisms of DHCA.

## Data Availability

All data generated or analyzed during this study are included in this published article.

## Abbreviations

ALT: Alanine aminotransferase
ARDS: Acute respiratory distress syndrome
AST: Aspartate aminotransferase
BUN: Blood urea nitrogen
CPB: Cardiopulmonary bypass
Cr: Creatinine
DHCA: Deep hypothermic circulatory arrest
LDH: Lactate dehydrogenase
HR: Heart rate
MAD: Malondialdehyde
MAP: Mean arterial pressure
MPO: Myeloperoxidase
SD rats: Sprague‒Dawley rats
SOD: Superoxide dismutase

## References

[1] Groom RC, Akl BF, Albus R, Lefrak EA (1996). Pediatric cardiopulmonary bypass: a review of current practice. Int Anesthesiol Clin.

[2] Buggeskov KB, Grã¸Nlykke L, Risom EC, Wei ML, Wetterslev J. Pulmonary artery perfusion versus no perfusion during cardiopulmonary bypass for open heart surgery in adults. Cochrane Database Syst Rev. 2018;2(3):CD011098. 10.1002/14651858.CD011098.pub2 PMC6491280 29419895

[3] Chau KH, Ziganshin BA, Elefteriades JA (2013). Deep hypothermic circulatory arrest: real-life suspended animation. Prog Cardiovasc Dis.

[4] Gutsche JT, Ghadimi K, Patel PA, Robinson AR, Lane BJ, Szeto WY (2014). New frontiers in aortic therapy: focus on deep hypothermic circulatory arrest. J Cardiothorac Vasc Anesth.

[5] Yang CL, Tsai PS, Huang CJ (2008). Effects of dexmedetomidine on regulating pulmonary inflammation in a rat model of ventilator-induced lung injury. Acta Anaesthesiol Taiwan.

[6] Roekaerts PM, Prinzen FW, De Lange S (1996). Beneficial effects of dexmedetomidine on ischaemic myocardium of anaesthetized dogs. Br J Anaesth.

[7] Paparella D, Yau TM, Young E (2002). Cardiopulmonary bypass induced inflammation: pathophysiology and treatment. An update. Eur J Cardiothorac Surg.

[8] Moore NM, Proia LA (2018). Severe acute respiratory distress syndrome in a liver transplant patient. Med Mycol Case Rep.

[9] Groth KE, Kelly TC, Westerbeck TL, Blick G. Treatment of hepatitis C using hyperthermia. 2001.

[10] Liao X, Fan S, Li Z, Chen J, Niu H, He Y (2006). Application of cardiopulmonary bypass during extended resection of locally advanced lung cancer. Acta Academiae Medicinae Militaris Tertiae.

[11] Schlensak C, Doenst T, Beyersdorf F (2000). Lung ischemia during cardiopulmonary bypass. Ann Thorac Surg.

[12] Sun H, Ross DA (2012). Fatal hemorrhagic infarction of posterior fossa meningioma during cardiopulmonary bypass. Ann Thorac Surg.

[13] Nollert G, Reichart B (2001). Cardiopulmonary bypass and cerebral injury in adults. Shock.

[14] Huffmyer JL, Groves DS (2015). Pulmonary complications of cardiopulmonary bypass. Best Pract Res Clin Anaesthesiol.

[15] Boshkov LK, Kirby A, Shen I, Ungerleider RM (2006). Recognition and management of heparin-induced thrombocytopenia in pediatric cardiopulmonary bypass patients. Ann Thorac Surg.

[16] Oktar BK, Gulpinar MA, Bozkurt A, Ghandour S, Cetinel S, Moini H (2002). Endothelin receptor blockers reduce I/R-induced intestinal mucosal injury: role of blood flow. Am J Physiol Gastrointest Liver Physiol.

[17] Abu-Omar Y, Ratnatunga C (2006). Cardiopulmonary bypass and renal injury. Perfusion.

[18] Tabayashi K (2005). Influences of cardiopulmonary bypass in elderly patients. Kyobu Geka.

[19] Schoenberg MH, Poch B, Younes M, Schwarz A, Baczako K, Lundberg C (1991). Involvement of neutrophils in postischaemic damage to the small intestine. Gut.

[20] Mayumi H, Zhang QW, Nakashima A, Masuda M, Kohno H, Kawachi Y (1997). Synergistic immunosuppression caused by high-dose methylprednisolone and cardiopulmonary bypass. Ann Thorac Surg.

[21] Tofukuji M, Stahl GL, Metais C, Tomita M, Agah A, Bianchi C (2000). Mesenteric dysfunction after cardiopulmonary bypass: role of complement C5a. Ann Thorac Surg.

[22] Ohri SK, Velissaris T (2006). Gastrointestinal dysfunction following cardiac surgery. Perfusion.

[23] Reed H, Berg KB, Janelle GM (2014). Aortic surgery and deep-hypothermic circulatory arrest: anesthetic update. Semin Cardiothorac Vasc Anesth.

[24] Salah M, Sutton R, Tsarovsky G, Djuric M (2005). Temperature inaccuracies during cardiopulmonary bypass. J Extra Corpor Technol.

[25] Chen Y, Liu J, Ji B, Tang Y, Wu A, Wang S (2012). The optimal flow rate for antegrade cerebral perfusion during deep hypothermic circulatory arrest. Artif Organs.

[26] Wang Q, Yang J, Long C, Zhao J, Li Y, Xue Q (2012). Hyperoxia management during deep hypothermia for cerebral protection in circulatory arrest rabbit model. ASAIO J.

[27] Li T, Wu W, You Z, Zhou R, Li Q, Zhu D (2012). Alternative use of isoflurane and propofol confers superior cardioprotection than using one of them alone in a dog model of cardiopulmonary bypass. Eur J Pharmacol.

[28] Waterbury T, Clark TJ, Niles S, Farivar RS (2011). Rat model of cardiopulmonary bypass for deep hypothermic circulatory arrest. J Thorac Cardiovasc Surg.

[29] Engels M, Bilgic E, Pinto A, Vasquez E, Wollschlager L, Steinbrenner H (2014). A cardiopulmonary bypass with deep hypothermic circulatory arrest rat model for the investigation of the systemic inflammation response and induced organ damage. J Inflamm (Lond).

[30] Lebreton G, Tamion F, Bessou JP, Doguet F (2012). Cardiopulmonary bypass model in the rat: a new minimal invasive model with a low flow volume. Interact Cardiovasc Thorac Surg.

[31] Samarska IV, Henning RH, Buikema H, Bouma HR, Houwertjes MC, Mungroop H (2013). Troubleshooting the rat model of cardiopulmonary bypass: effects of avoiding blood transfusion on long-term survival, inflammation and organ damage. J Pharmacol Toxicol Methods.

[32] Sever K, Ozbek C, Goktas B, Bas S, Ugurlucan M, Mansuroglu D (2014). Gastrointestinal complications after open heart surgery: incidence and determinants of risk factors. Angiology.

[33] Hashemzadeh K, Hashemzadeh S (2012). Predictors and outcome of gastrointestinal complications after cardiac surgery. Minerva Chir.

[34] Rodriguez F, Nguyen TC, Galanko JA, Morton J (2007). Gastrointestinal complications after coronary artery bypass grafting: a national study of morbidity and mortality predictors. J Am Coll Surg.

[35] Viana FF, Chen Y, Almeida AA, Baxter HD, Cochrane AD, Smith JA (2013). Gastrointestinal complications after cardiac surgery: 10-year experience of a single Australian centre. ANZ J Surg.

[36] Gordan ML, Kellermann K, Blobner M, Nollert G, Kochs EF, Jungwirth B (2010). Fast rewarming after deep hypothermic circulatory arrest in rats impairs histologic outcome and increases NFkappaB expression in the brain. Perfusion.

[37] Ohri SK (1996). Systemic inflammatory response and the splanchnic bed in cardiopulmonary bypass. Perfusion.

[38] Doguet F, Tamion F, Le Guillou V, Bubenheim M, Thuillez C, Richard V (2012). Albumin limits mesenteric endothelial dysfunction and inflammatory response in cardiopulmonary bypass. Artif Organs.

[39] Abboud B, Daher R, Boujaoude J (2008). Acute mesenteric ischemia after cardio-pulmonary bypass surgery. World J Gastroenterol.

[40] Bassiouny HS (1997). Nonocclusive mesenteric ischemia. Surg Clin North Am.

[41] Ohri SK, Becket J, Brannan J, Keogh BE, Taylor KM (1994). Effects of cardiopulmonary bypass on gut blood flow, oxygen utilization, and intramucosal pH. Ann Thorac Surg.

[42] Doguet F, Litzler PY, Tamion F, Richard V, Hellot MF, Thuillez C, et al. Changes in mesenteric vascular reactivity and inflammatory response after cardiopulmonary bypass in a rat model. Ann Thorac Surg. 2004;77(6):2130–7; author reply 7. 10.1016/j.athoracsur.2003.10.034 15172281

[43] Sack FU, Hagl S. Extracorporeal circulation and intestinal microcirculation: pathophysiology and therapeutical options. An intravital microscopic study in a large animal model. Eur Surg Res. 2002;34(1–2):129–37. 10.1159/000048899 11867913

